# OAS3 is a Co-Immune Biomarker Associated With Tumour Microenvironment, Disease Staging, Prognosis, and Treatment Response in Multiple Cancer Types

**DOI:** 10.3389/fcell.2022.815480

**Published:** 2022-05-03

**Authors:** Xin-yu Li, Lei Hou, Lu-yu Zhang, Liming Zhang, Deming Wang, Zhenfeng Wang, Ming-Zhe Wen, Xi-tao Yang

**Affiliations:** ^1^ Department of Interventional Therapy, Shanghai Ninth People’s Hospital, Shanghai Jiao Tong University School of Medicine, Shanghai, China; ^2^ Department of Neurosurgery, Shanghai Ninth People’s Hospital, Shanghai JiaoTong University School of Medicine, Shanghai, China; ^3^ Jiading District Central Hospital Affiliated Shanghai University of Medicine and Health Sciences, Shanghai, China; ^4^ Department of Urologic Surgery, The First Affiliated Hospital of Zhengzhou University, Zhengzhou, China

**Keywords:** OAS3, pancancer analysis, biomarker, tumour microenvironment, prognosis carcinoma

## Abstract

2′,5′-oligoadenylate synthase (OAS) is a class of enzymes induced by interferons and mainly encoded by the OAS1, OAS2, and OAS3 genes, which activate the potential RNA enzymes to degrade viral mRNA, inhibit viral protein synthesis and promote apoptosis in virus-infected cells. *OAS3* is associated with breast cancer prognosis. However, the expression and prognosis of *OAS3* and tumour-infiltrating lymphocytes in pan-cancer remain unknown. In the present study, we have systematically investigated and confirmed the role of *OAS3* in tumour immune infiltration, immune escape, tumour progression, response to treatment, and prognosis of different cancer types using various bioinformatics methods. The findings suggest that *OAS3* is aberrantly expressed in almost all TCGA cancer types and subtypes and is associated with tumour staging, metastasis, and prognostic deterioration in different tumours. In addition, *OAS3* expression is associated with the prognosis and chemotherapeutic outcomes of various cancers. In terms of immune-infiltrating levels, *OAS3* expression is positively associated with the infiltration of immunosuppressive cells. These findings suggest that *OAS3* is correlated with prognosis and immune-infiltrating levels.

## Introduction

The development and progression of malignancy is a complex process involving several stages (Yang et al., 2019). Malignant tumours are heterogeneous and result from an accumulation of distinct genetic and epigenetic alterations (Lin et al., 2015). Several studies have suggested that genetic and epigenetic alterations can be functionally associated with carcinogenesis (Toyota and Suzuki, 2010; Coppedè et al., 2014; Grady et al., 2021). The tumor microenvironment (TME) is a complex cellular ecosystem in which tumor, stroma, and immune cells interact dynamically through secreted factors and physical interactions in a dynamic extracellular matrix (Grauel et al., 2020). The complexity of TME results in an interplay of various cellular signalling systems in which tumour cells infiltrate immune cells, making them dysfunctional, and hence unable to initiate any anti-tumour immune action (Jiang et al., 2015; Thommen and Schumacher, 2018). In addition, the immunosuppressive cellular component of TME may inhibit T-cell responses, antibody production and the induction of cytotoxic T lymphocytes, promoting tumour growth, impairing the immune response, and leading to treatment resistance (Liu and Cao, 2016; Monteran and Erez, 2019). Bioinformatics can accurately capture cell-type-specific profiles and cell–cell interactions at the tissue level, resulting in relevant genomic differences in the diagnosis, staging, prognosis, and therapeutic responses among various tumours.

2′-5′-oligoadenylate synthetase (OAS), an interferon-induced antiviral enzyme, is composed of OAS1, OAS2, OAS3, and OASL. OAS3 plays a critical role in antiviral action and signal transduction, and high OAS3 expression is associated with the poor prognosis of patients with breast cancer (Zhang and Yu, 2020). Owing to the complexity of tumorigenesis, pan-cancer analysis of the expression patterns of target genes and assessment of their correlation with clinical prognosis and potential molecular mechanisms are of great importance. In this study, we performed a pan-cancer analysis to examine the expression profiles of OAS3 in different cancer tissues and identify its underlying molecular mechanisms in the clinical prognosis of tumours.

## Materials and Methods

### Data Collection and Evaluation of OAS3 Expression in Pan-Cancer

**FIGURE 1:**
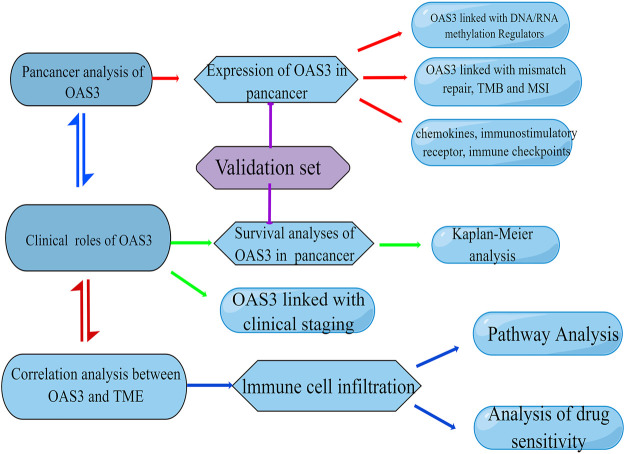
Flowchart of the study. This image was drawn by Figdraw (www.figdraw.com).

RNA sequence data, survival data, and clinicopathologic characteristics of the 33 cancers were obtained from the UCEC online database (https://xena.ucsc.edu/), which was obtained from the TCGA database (Tomczak et al., 2015). Using the rma function in the R package, the whole dataset was filtered, and missing and duplicate results were removed and converted to log2 (TPM + 1). OAS3 sequencing data were obtained from the GTEx Project and Broad Institute CCLE database to analyse differences between tumours and adjacent normal tissues. In addition, 36 patients with liver hepatocellular carcinoma (LIHC), 30 patients with lung adenocarcinoma (LUAD), and 30 patients with kidney renal papillary cell carcinoma (KIRP) were recruited from the First Affiliated Hospital of Zhengzhou University and Jiading District Central Hospital Affiliated Shanghai University of Medicine as the validation cohort. The basic clinical characteristics of patients are shown in Table 1, Table 2, and Table 3. All patients provided written informed consent for the data to be included in the study. The study flowchart is presented in Figure 1.

**TABLE 1:** Basic clinical characteristics of LIHC patients in validation cohort.

Characteristic	Levels	Overall
n		36
T stage, n (%)	T1	18 (50%)
	T2	7 (20%)
	T3	7 (20%)
	T4	4 (10%)
N stage, n (%)	N0	32 (90%)
	N1	4 (10%)
M stage, n (%)	M0	32 (90%)
	M1	4 (10%)
Pathologic stage, n (%)	Stage I	18 (50%)
	Stage II	9 (25%)
	Stage III	4(10%)
	Stage IV	9 (13%)
Age, n (%)	<=60	18 (50%)
	>60	
Gender, n (%)	Female	15 (40%)
	Male	21(60%)
Age, median (IQR)		55 (50, 61)

**TABLE 2:** Basic clinical characteristics of LUAD patients in validation cohort.

Characteristic	Levels	Overall
n		30
T stage, n (%)	T1	9 (30%)
	T2	15(50%)
	T3	3(10%)
	T4	3(10%)
N stage, n (%)	N0	21(70%)
	N1	5 (16%)
	N2	3 (10%)
	N3	1 (4%)
M stage, n (%)	M0	27(90%)
	M1	3 (10%)
Pathologic stage, n (%)	Stage I	15(50%)
	Stage II	6 (20%)
	Stage III	5 (16%)
	Stage IV	4 (14%)
Gender, n (%)	Female	15(50%)
	Male	15(50%)
Age, n (%)	<=65	15(50%)
	>65	15(50%)
Age, median (IQR)		60 (53, 71)

**TABLE 3:** Basic clinical characteristics of KIRP patients in validation cohort.

Characteristic	Levels	Overall
n		30
Pathologic T stage, n (%)	T1	21 (70%)
	T2	3 (10%)
	T3	3 (10%)
	T4	3 (10%)
Pathologic N stage, n (%)	N0	18 (60%)
	N1	9 (30%)
	N2	3 (10%)
Pathologic M stage, n (%)	M0	27 (90%)
	M1	3 (10%)
Pathologic stage, n (%)	Stage I	18 (60%)
	Stage II	3 (10%)
	Stage III	6 (20%)
	Stage IV	3 (10%)
Gender, n (%)	Female	9 (30%)
	Male	21 (70%)
Age, n (%)	<=60	15 (50%)
	>60	15 (50%)
Age, median (IQR)		62 (55, 70)

### Prognostic Significance of OAS3

We used four prognostic indicators (including overall survival [OS], disease-specific survival [DSS], disease-free survival [DFS], and disease progression-free survival [PFS]) and investigated the relationship between *OAS3* expression and the prognosis of patients with 33 cancers using forest plots. Survival results were summarised using “forestplot” (R package). Patients were divided into high and low *OAS3* expression groups based on the median *OAS3* expression. Kaplan–Meier survival analysis was conducted using the R packages “survminer” and “survival” to examine differential survival outcomes between the two groups.

### Correlation Analysis of OAS3 Expression With Microsatellite Instability and Tumour Mutational Burden

Tumour mutational burden (TMB) is defined as the total number of mutations per million bases in the coding region of the exons of genes encoding specific tumour cell proteins, including insertions, substitutions, deletions, and other forms of mutations (Alexandrov et al., 2013). It is also an emerging biomarker for tumour immunotherapy prediction and may help to predict the benefits of immunotherapy in certain tumours. Microsatellite instability (MSI) is characterised by a genetic change. During the proliferation of normal cells, an intact DNA mismatch repair (MMR) system detects replication errors in microsatellite sequences in a timely manner and corrects them quickly so that the sequences are replicated with high fidelity, thus maintaining microsatellite stability. Owing to defective DNA MMR during tumorigenesis in certain tumours, errors in microsatellite sequence replication cannot be detected promptly, leading to the insertion or deletion of repetitive units and changes in microsatellite sequence length, eventually leading to MSI (Ellegren, 2004). Several clinical trials, retrospective studies and meta-analyses have confirmed that MSI is strongly associated with tumour prognosis (Petrelli et al., 2019). In this study, gene mutation data of 33 cancer types were obtained from TCGA database of UCSC Xena. TMB was calculated for each sample using R. The correlation of OAS3 expression with TMB and MSI was analysed via Spearman’s correlation test, and the R package “fmsb” was used to visualise the results.

### Correlation Analysis of OAS3 Expression With TME

TME is critical for the regulation of cancer development and therapy (Zhou et al., 2019). It contains stromal, tumour, and immune cells (Luo and Vögeli, 2020). The number of stromal and immune cells in TME affects many aspects of cancer growth and development. The “ESTIMATE” R package was used to assess immune infiltration (based on the ImmuneScore, StromalScore, and ESTIMATEScore) using the transcriptome data (Chen et al., 2021a). Subsequently, we analysed the association between OAS3 and TME using R.

### Analysis of Tumour Immune Cell Infiltration

We used TIMER2, Xcell, CIBERSORT, and ImmuCellAI to analyse the correlation between OAS3 expression and infiltration of various immune cell types. The TIMER2 database contains information on 32 cancers and 10,897 tissues from TCGA database, which allows systematic analysis of the correlation between one or more tumours and immune cell infiltration as well as the correlation between the expression of relevant genes in tumour tissues and the prognosis, mutation and copy number of patients (Ju et al., 2020). ImmuCellAI (http://bioinfo.life.hust.edu.cn/web/ImmuCellAI/) is a powerful and unique method for accurately screening tumour immune function using 24 different types of immune cells, including T cells (Huang et al., 2021). Furthermore, the XCell algorithm was used to examine several features of tumours, including the composition of infiltrating immune cells, based on the gene expression data (Schulze et al., 2020). In addition, the CIBERSORT algorithm was used to identify immune cell infiltration signatures using the R package “cibersort” (Wu et al., 2020).

### Correlation of OAS3 Expression With Immune Checkpoint-Related Genes and Immune Neoantigens

Immune checkpoints refer to a subset of inhibitory signalling pathways involved in the immune response (Yuan et al., 2020). Abnormal expression of immune checkpoint-related genes is associated with tumorigenesis (Liu et al., 2021a). We examined the association of OAS3 with 47 immune checkpoint-associated genes in 33 cancers using Pearson correlation analysis. Neoantigens are abnormal proteins derived from “nonsynonymous mutations” from biological events such as point mutations, deletion mutations, and gene fusions and are specific to tumour cells (Liao and Zhang, 2021; Xu et al., 2021). The immune activity of tumour neoantigens can be used to design and synthesise neoantigen vaccines according to the conditions of the bulge of the swollen cell; these vaccines can be used to immunise patients to achieve therapeutic effects (Chen et al., 2021b). We counted the number of neoantigens in each tumour sample and used Spearman’s correlation test to investigate the relationship between OAS3 and the number of antigens.

### Correlation of OAS3 Expression With the Expression of DNA MMR Genes, RNA Methylation-Related Genes and DNA Methyltransferase

MMR is a critical post-replicative DNA repair process, which is essential for maintaining genomic integrity (Popp and Bohlander, 2010). Defects in the MMR system lead to genetic instability referred to as MSI (Alhopuro et al., 2012). DNA methylation is a chemical modification of DNA that can change genetic performance without changing the DNA sequence (Alhopuro et al., 2012). RNA methylation is one of the most important post-transcriptional epigenetic RNA modifications (Tian et al., 2021). The most commonly used RNA modifications are m6A, m1A, and m5C, which play a key role in the progression of cancers, including growth and invasion (Mcelhinney et al., 2020). In this study, we examined the relationship between OAS3 expression and the abovementioned genes using the R package “RColorBrewer”.

### Correlation of OAS3 Expression With Drug Sensitivity

The relationship between OAS3 and IC50 of drugs was analysed based on the GDSC2 data. In addition, we compared the drug sensitivity of the OAS family using the Cancer Therapeutics Response Portal 21 (CTRP, http://portals.broadinstitute.org/ctrp/). The likelihood of an immunotherapy response was estimated using the TIDE algorithm (Khanna et al., 2019; Yang et al., 2020).

### Quantitative Real-Time PCR

Total RNA was extracted from the target tissue samples and thoroughly ground in a mortar under liquid nitrogen. To lyse the cells, 1 ml of Trizol reagent (Life Technology, Grand Island, NY, United States) was added and the sample was incubated for 15 min at room temperature on a shaker. To assess the mRNA expression level, the RevertAid First Strand cDNA Synthesis Kit (Thermo Scientific, Lithuania) was used to synthesis the first-strand cDNA. Quantitative PCR was performed using Roche LightCycler^®^ 480 Real-Time PCR System with SYBR^®^ Green qPCR mix 2.0 kit. The primers used in this study were obtained from TsingKe biological technology (Nanjing, China), including *OAS3* (forward 5′- CAC​CGG​CGA​TGC​CCG​CAT​CTC​ACT​G -3′, reverse 5′- AAA​CCA​GTG​AGA​TGC​GGG​CAT​CGC​C-3′). The relative mRNA levels were calculated by the 2-ΔΔCt method.

### Western Blot

Western blot was performed to determine the protein expression level. The protein samples were denatured for 5 min at 95°C in a sample buffer and separated by SDS–PAGE. Western blot analysis was performed using antibodies against mouse monoclonal antibody-anti-human OAS3, and mouse monoclonal antibody-anti-human β-actin (Santa Cruz Biotechnology), followed by incubation with horseradish peroxidase (HRP)-coupled mouse secondary antibody (1:10,000). The blots were re-probed with a β-actin antibody (BD Bioscience, United States), and the signals were quantified using an image analyzer (UVtec, United Kingdom). The data were shown as percentages of the normalized control signal.

## Results

### Expression Levels of OAS3 in Various Normal and Cancerous Tissues

**FIGURE 2:**
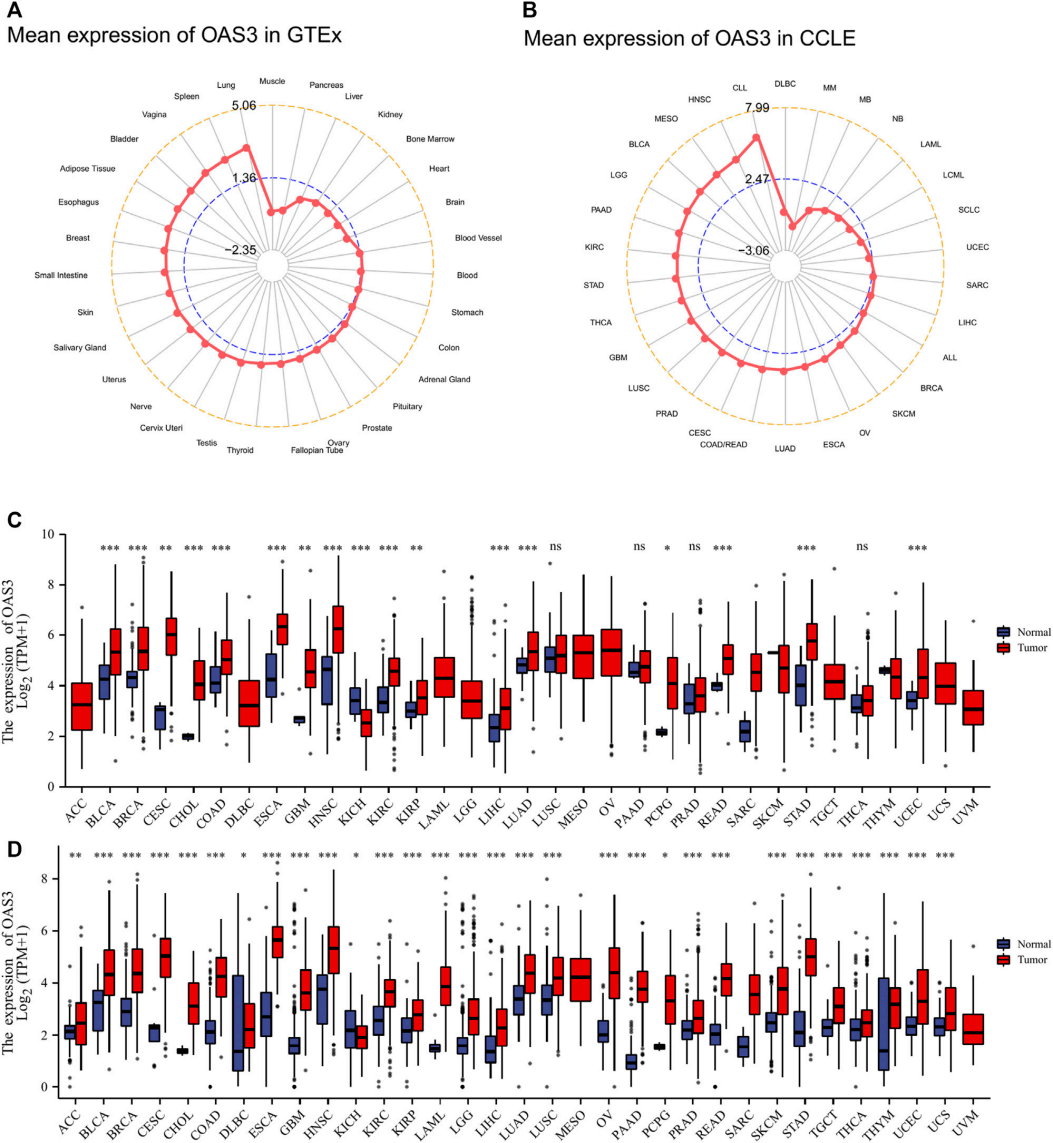
**(A)**: OAS3 mRNA expressions in various normal human tissues from the GTEx database. OAS3 is most highly expressed in lung and least expressed in muscle. **(B)**: OAS3 mRNA expressions from the CCLE database. The results showed that OAS3 was highly expressed in most tumors. **(C)**: Boxplots showing differential OAS3 expression levels (log2TPM + 1) between tumor and adjacent normal tissues across TCGA database. *p < 0.05; **p < 0.01; ***p < 0.001. **(D)**: Boxplots showing differential OAS3 expression levels (log2TPM + 1) between tumor and adjacent normal tissues across TCGA and GTEx database. *p < 0.05; **p < 0.01; ***p < 0.001.

Data from the GTEx database showed that *OAS3* was abundantly expressed in various normal tissues, with the highest and lowest expression observed in the lung and muscle, respectively (Figure 2A). In addition, the expression of *OAS3* was higher in various cancer cell lines in the Cancer Cell Line Encyclopedia (CCLE) database than in normal tissue (Figure 2B). In TCGA data, differences in *OAS3* expression were significant among 17 of the 33 cancer types analysed (except for KICH, in which *OAS3* expression was lower than that in most tumour tissues) (Figure 2C). However, when the GTEx and TCGA data were combined, the difference was significant among 29 of the 33 cancers, and *OAS3* expression was lower in KICH than in normal tissues (Figure 2D). To investigate the intracellular localisation of *OAS3*, we assessed the distribution of *OAS3* in the endoplasmic reticulum (ER) and microtubules of A431, A549, and U-2 osteosarcoma (OS) cells using an indirect immunofluorescence assay. We observed that *OAS3* colocalised with ER and microtubule markers in A431, A549 and U-2 OS cells, suggesting the subcellular localisation of *OAS3* in ER and microtubules. However, *OAS3* exhibited no overlap with the nucleus of A431, A549, and U-2 OS cells (Supplementary Figure S1).

### Relationship of OAS3 With Clinical Staging and Prognosis

**FIGURE 3:**
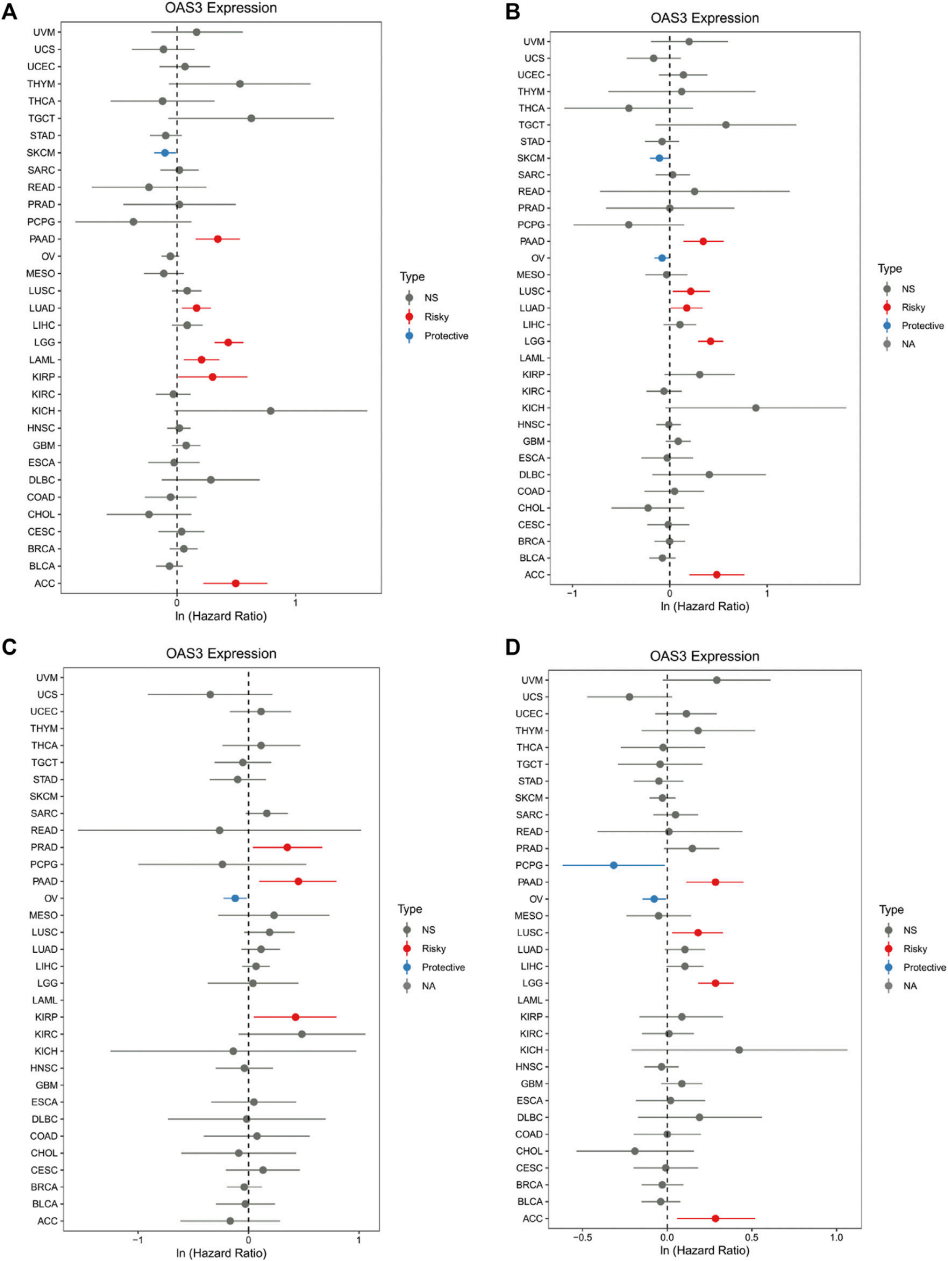
Forest plot of the association of OAS3 expression with OS **(A)**, DSS **(B)**, DFS **(C)**, and PFS **(D)**. **(A)**: OAS3 is highly expressed in patients with PAAD, LUAD, LGG, LAML, KIRP, ASS with poor OS. In SKCM, high expression of OAS3 is a protective factor for OS. **(B)**: In PAAD, LUSC, LUAD, LGG, ACC, patients with high OAS3 expression showed shorter DSS than those with low expression. in SKCM, OV, the opposite effect of OAS3 was observed. **(C)**: OAS3 was highly expressed in PAAD, PRAD, and KIRP patients with poor DFS. In contrast, in OV, high OAS3 expression was associated with longer DFS. **(D)**: Patients with high OAS3 expression in PAAD, LUSC, LGG, and ACC had shorter DFS than those with low expression. In PCPG, the opposite role of OAS3 was observed.

We examined the relationship between *OAS3* expression and different tumour stages and found that DLBC, HNSC, KIRC, LIHC, LUSC, MESO, OV, PAAD, LUAD, SKCM, and UCS were positively correlated with the expression of *OAS3*. This finding suggests that *OAS3* plays an important role in tumorigenesis (Supplementary Figure S2). Furthermore, we investigated the relationship between *OAS3* expression and the prognosis of 33 cancers. According to the Cox proportional hazards model, *OAS3* expression was positively correlated with OS in patients with PAAD, LUAD, LGG, LAML, KIRP, and ACC and negatively correlated with OS in patients with SKCM (Figure 3A). In addition, we analysed the DSS data and found a positive correlation between *OAS3* expression and prognosis in patients with PAAD, LUSC, LUAD, LGG, and ACC. However, *OAS3* expression was negatively correlated with the prognosis of SKCM and OV (Figure 3B). Based on the correlation between *OAS3* expression and DFS, we identified *OAS3* as a prognostic risk factor for PRAD, PAAD, and KIRP but as a protective factor for OV (Figure 3C). Similarly, high *OAS3* expression was associated with worse PFS in PAAD, LUSC, LGG, and ACC (Figure 3D). Furthermore, Kaplan–Meier analysis showed that high *OAS3* expression was associated with worse OS in ACC, DLBC, KICH, KIRP, LAML, LGG, LUAD, and PAAD but with better OS in MESO (Supplementary Figure S3). High *OAS3* expression was correlated with worse DFS in four types of tumours, including KIRP and PAAD (Supplementary Figure S4). In seven types of tumours, including ACC and DLBC, patients with high *OAS3* expression had worse DSS (Supplementary Figure S5). These findings suggest that *OAS3* is an oncogene that is associated with tumour progression, can help to predict survival in patients with various tumours and is a potential biomarker for tumour prognosis, especially for the prognosis of PAAD, LUAD, KIRP, and UVM.

### Validation of the Expression and Prognostic Role of OAS3

**FIGURE 4:**
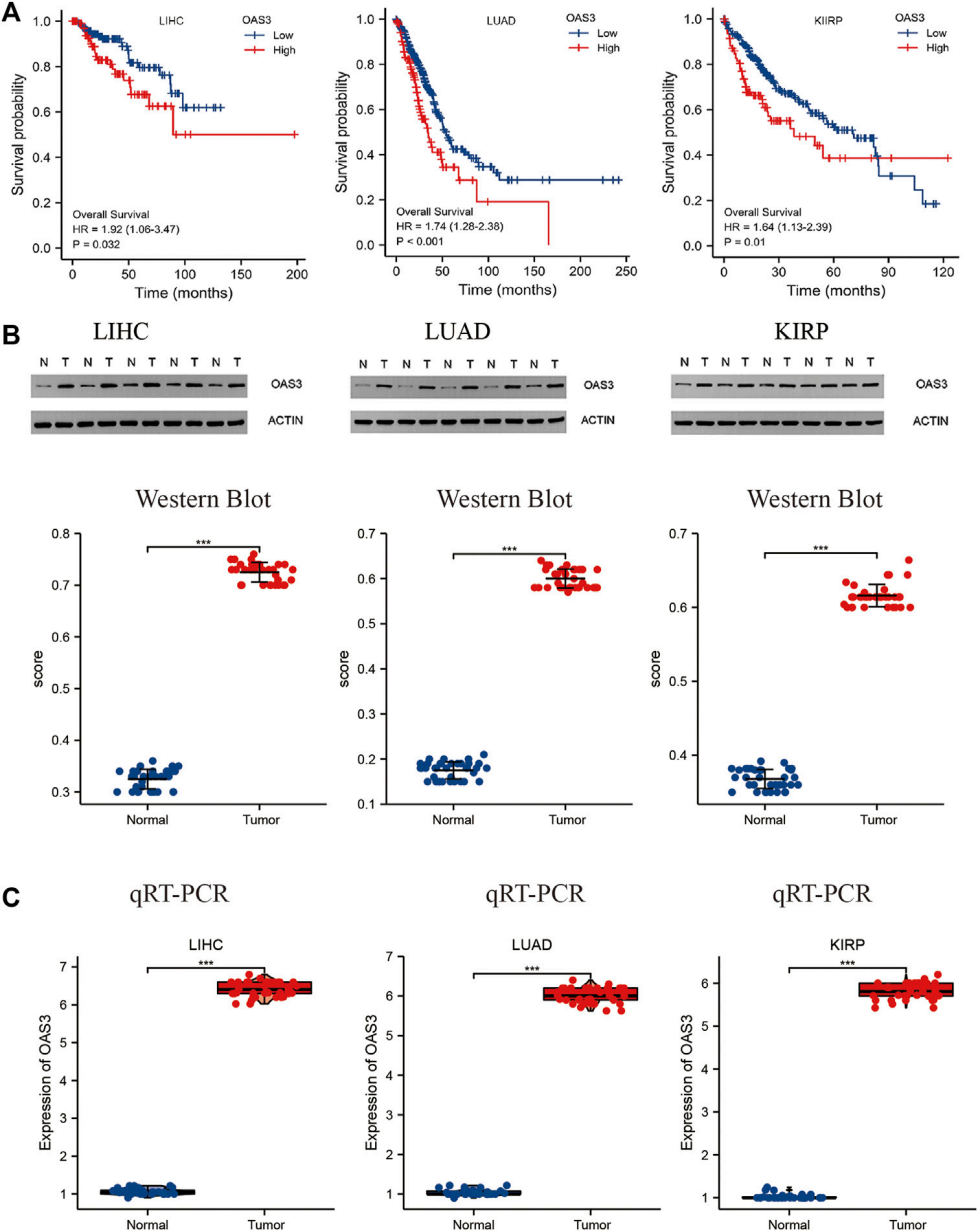
**(A)** Relationship between OAS3 and OS of LIHC, LUAD, KIRP in validation cohort. The results showed that patients with high OAS3 expression had a poor prognosis in the three tumors mentioned above. **(B,C)**: Results of Western Blot **(B)** and qRT-PCR **(C)** showed a clear overexpression in protein expression levels of OAS3 in validation cohort.

To substantiate the conclusion of the data analysis, we validated *OAS3* expression in 36 patients with LIHC, 30 patients with LUAD and 30 patients with KIRP and performed survival analysis in conjunction with clinical trials. As shown in Figure 4A, patients with high *OAS3* expression in the validation group had a poorer prognosis in patients with LUAD, KIRP; this is consistent with our findings in the TCGA cohort. Also, we found a poorer prognosis for patients with high *OAS3* expression in LIHC in the validation group. Meanwhile, OAS3 was detected using qRT-PCR and western blotting. The results of western blotting was consistent with those of qRT-PCR: *OAS3* was highly expressed in tumor tissues in LIHC, LUAD, KIRP, and low in normal tissues (Figures 4B, C). TCGA cohort results are mostly consistent with our findings. This ensured breadth and thereby enhanced credibility in the data.

### Correlation of OAS3 With TME and Immune-Infiltrating Cells

**FIGURE 5:**
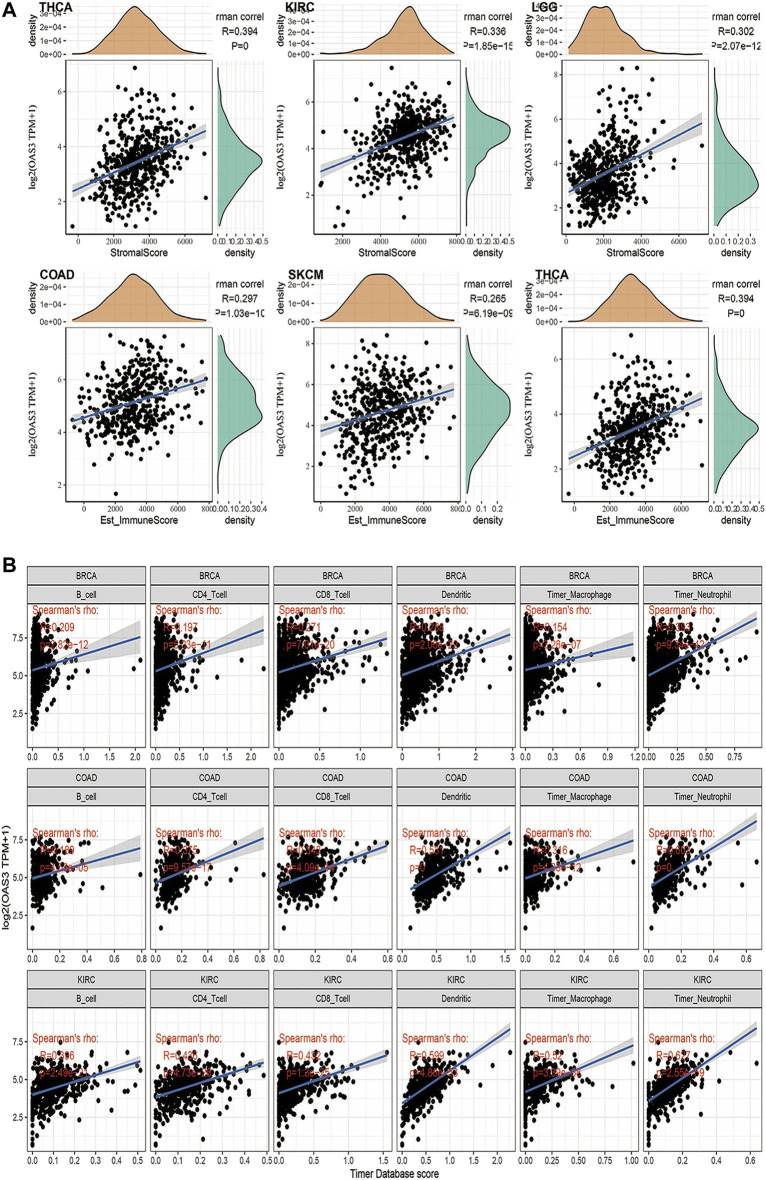
**(A)** Among the 33 tumors, the top 3 tumors with the highest stromal scores were THCA, KIRC, LGG; the highest immune scores were COAD, SKCM, THCA; the highest ESTIMATEScore was COAD, SKCM,THCA. **(B)** TIMER analyzed relationship between OAS3 expression and the abundance of tumor-infiltrating immune cells in BRCA, COAD, KIRC. The results showed a positive correlation between OAS3 expression and immune cell infiltration.

We evaluated the correlation between OAS3 and immune and stromal scores. As shown in Supplementary Figure S6, immune scores, ESTIMATEScore and stromal scores were significantly correlated with OAS3 expression in 20, 25, and 20 of the 33 cancers, respectively. Figure 5A shows the top three significant genes in each score, with COAD having the highest immune score and ESTIMATEScore and THCA having the highest substrate score. The results showed that *OAS3* expression strongly correlates with the degree of immune infiltration in different cancer types. Therefore, we investigated the relationship between *OAS3* and immune infiltrating cells in 33 cancer types using the TIMER database. The top three significant cell types are shown in Figure 5B, demonstrating that *OAS3* correlates significantly with tumor purity and with six types of immunoinfiltrated cells, including CD8^+^ T cells, CD4^+^ T cells, B cells, dendritic cells, and macrophages in KIRC, COAD and BRCA. Based on the results of immune analysis, *OAS3* was related to poor prognosis in some tumours and might affect immune activities. Therefore, we examined the correlation between *OAS3* and immune-related cells using Xcell, TIMER2, CIBERSORT and ImmuCellAI to validate the results and found that *OAS3* was significantly correlated with neutrophils and macrophages (Supplementary Figures S7A–C). In addition, cancer-associated fibroblasts, a type of immunosuppressive cells, had a strong positive correlation with *OAS3* expression. The data from the ImmuCellAI database further suggested a significant positive correlation between *OAS3*and the immunosuppressive cells iTregs and nTregs (Supplementary Figure S7D). Therefore, *OAS3* may affect tumour progression through macrophages, iTregs, CAFs, and other immune cells.

### Correlation of OAS3 Expression With Immune Checkpoint Genes and the Number of Immune Neoantigens Implicates OAS3 in the Tumour Immune Response

**FIGURE 6:**
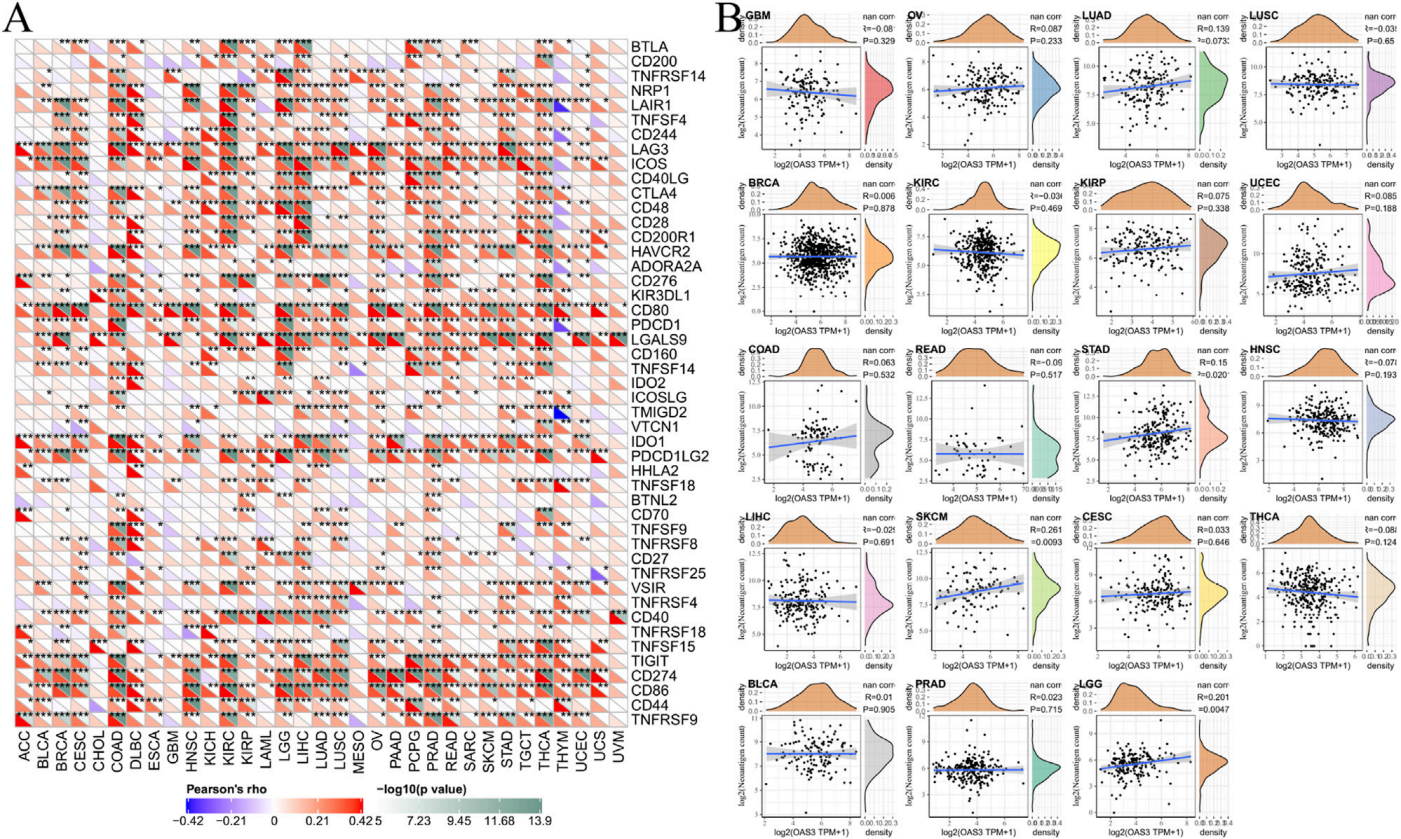
**(A)** The correlation between OAS3 expression and immune checkpoint genes in pan-cancer. Each square corresponded to the correlation between OAS3 expression and the expression of one immune checkpoint gene in a particular tumor. The upper triangle of each square represented the magnitude of the *p* value of the correlation test, and the lower triangle represented the correlation coefficient (∗*p* < 0.05, ∗∗*p* < 0.01, ∗∗∗*p* < 0.001). **(B)** The correlation between OAS3 expression and neoantigens.

The correlation between *OAS3* expression and 47 immune checkpoint genes in pan-cancer is shown in Figure 6A. In diverse cancer types, the correlation between *OAS3* and the expression of checkpoint genes indicated a high correlation with TNF-related immune genes including TNFRSF14, TNFRSF8, TNFRSF25, TNFRSF4, TNFRSF18, TNFSF15, and TNFRSF9. The expression of *OAS3* was positively correlated with that of immune checkpoint-related genes in most tumours, suggesting that *OAS3* is involved in the regulation of tumour immune response through the regulation of immune checkpoint activity. Therefore, *OAS3* may provide some help for tumour immunotherapy, thus facilitating the spread of tumours. The correlation between *OAS3* expression and neoantigens is shown in Figure 6B. A significant positive correlation was found between *OAS3* expression and the number of neoantigens in LGG, SKCM, and STAD.

### OAS3 Expression Was Significantly Correlated With TMB and MSI

**FIGURE 7:**
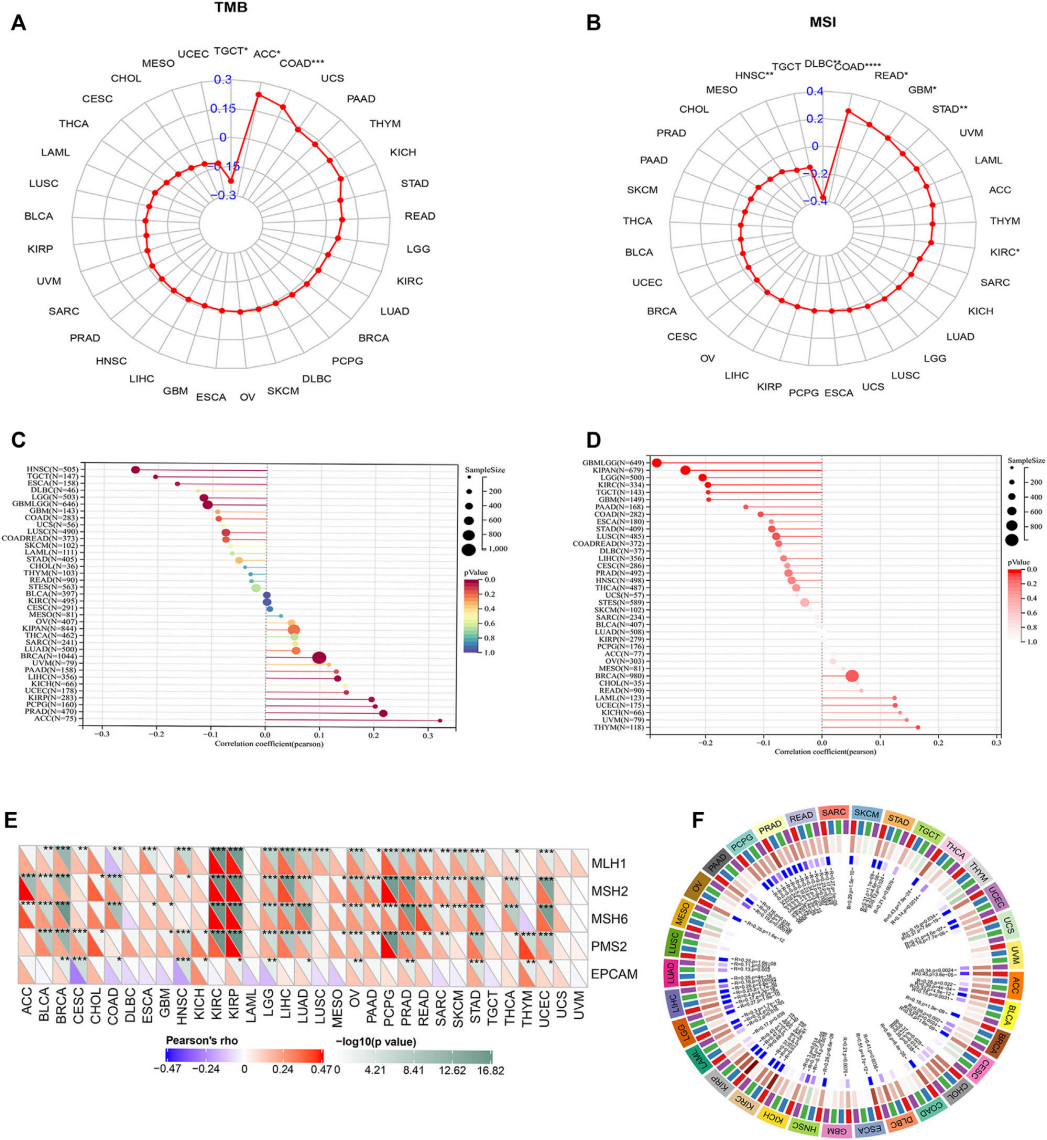
**(A)** Radar plot showing the correlation between OAS3 expression and TMB in pan-cancer. The blue number represents Spearman’s correlation coefficient. ∗*p* < 0.05, ∗∗*p* < 0.01, ∗∗∗*p* < 0.001. **(B)** Radar plot showing the correlation between OAS3 expression and MSI in pan-cancer. The blue number represents Spearman’s correlation coefficient. ∗*p* < 0.05, ∗∗*p* < 0.01, ∗∗∗*p* < 0.001. **(C)** The correlation between OAS3 expression and HBD in pan-cancer. The size of the circle represents the number of samples. The colors represent significant *p*-values (*p* < 0.05) for each section; red low *p*-value, blue high *p*-value. **(D)** Relationship between OAS3 and heterogeneity. The size of the circle represents the number of samples. Color represents *p*-value, the darker the color the more significant the result. **(E)** OAS3 was closely associated with DNA MMR genes. The upper triangle of each square represented the magnitude of the *p* value of the correlation test, and the lower triangle represented the correlation coefficient. *p < 0.05; **p < 0.01; ***p < 0.001. **(F)** OAS3 was positively correlated with four major DNA methyltransferases including DNMT1, DNMT2, DNMT3A, and DNMT3B in most cancer types.

We obtained *OAS3* mutation data of various tumours from the UCSC Xena database. *OAS3* mRNA was found to be significantly mutated in TGCT, ACC, COAD, and other tumours (Figure 7A), suggesting that mutated *OAS3* plays a key role in promoting the development of these tumours (Figure 7B). HRD produces specific, quantifiable, and stable genomic alterations, and the HRD status is a key indicator of treatment choice and prognosis in various tumours. Clinical studies have confirmed that the HRD status is highly correlated with sensitivity to platinum-based chemotherapeutic agents and PARP inhibitors (Min et al., 2020). We found that *OAS3* was positively correlated with HRD in ACC, PRAD, and KIRP (Figure 7C), and the heterogeneity of THYM, UVM, and KICH increases with an increase in OAS3 expression (Figure 7D).

### Coexpression of OAS3 With Some Specific Genes

Based on the association between *OAS3* expression and the mutational markers TMB and MSI, the relationship between *OAS3* expression and oncogenic processes was further investigated. We found that *OAS3* was closely associated with DNA MMR genes, showing a positive correlation with MLH1, MSH2, MSH6, and PMS2 and a negative correlation with EPCAM in most tumours (Figure 7E). In addition, *OAS3* was positively correlated with four major DNA methyltransferases including DNMT1, DNMT2, DNMT3A, and DNMT3B in most cancer types (Figure 7F). RNA methylation is a post-transcriptional modification that widely exists in eukaryotes and prokaryotes. We found that *OAS3* had a significant positive correlation with RNA methylation-related genes (m1A, m5C, and m6A) in most tumours (Supplementary Figure S8A). In addition, a positive correlation was found between *OAS3* and four immune pathway-related genes [receptor (Chen et al., 2021a), MHC (Schulze et al., 2020), immunoinhibitors (Liu et al., 2021a) and immunostimulators ^[46]^] in many tumour types (Supplementary Figure S8B).

### Analysis of Drug Sensitivity

We analysed 198 drugs and found that *OAS3* shared the most evident positive correlation with BI-2536, GSK269962A, vorinostat, sorafenib, BMS-754807, and nutlin-3a (−), indicating that high *OAS3* expression may lead to drug resistance. *OAS3* had the strongest negative correlation with trametinib, sapitinib, SCH772984, selumetinib, and dasatinib (Supplementary Figure S9A). A bubble plot demonstrating the correlation between the sensitivity of drugs in the CTRP database and mRNA expression of *OAS3* is shown in Supplementary Figure S9B.

### OAS3 Was Correlated With Immunotherapeutic Responses

**FIGURE 8:**
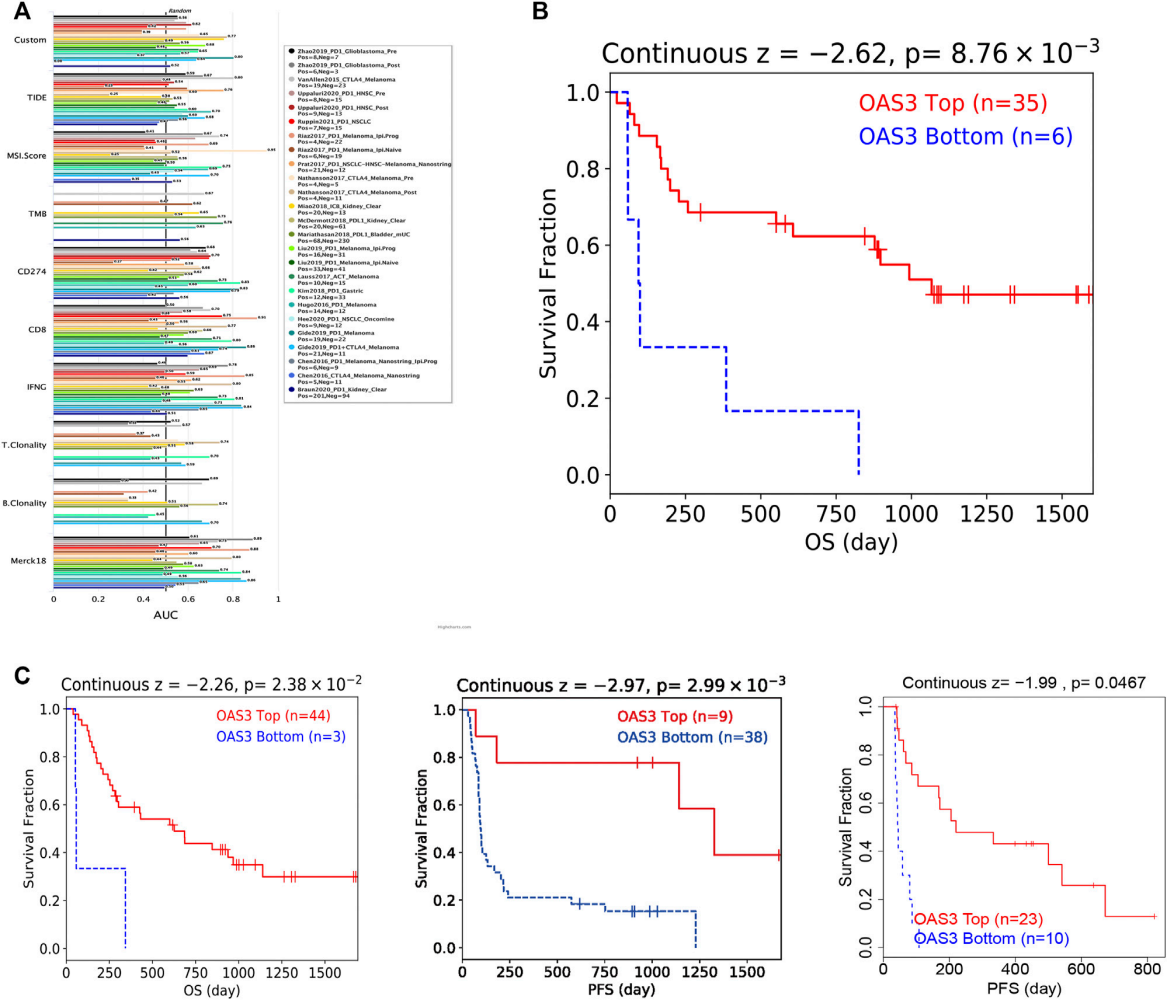
**(A)** Bar plot showing the biomarker relevance of OAS3 compared to standardized cancer immune evasion biomarkers in immune checkpoint blockade (ICB) sub-cohorts. The area under the receiver operating characteristic curve (AUC) was used to assess the predictive efficacy of test biomarkers for determining IBC response to TCGA for different cancer types. **(B,C)** Kaplan-Meier curves of survival ratios as a measure of the immunotherapeutic response (immune checkpoint blockade) between cancer cohorts with high and those with low expression levels of OAS3.

We assessed the reliability of *OAS3* as a biomarker by comparing it with standardised biomarkers for predicting response and OS in the IBC subpopulation. We found that the area under the receiver operating characteristic curve (AUC) of *OAS3* alone was greater than 0.5 in 16 of 23 IBC subgroups (Figure 8A), and that the predictive value of *OAS3* alone was higher than that of TMB, T. clonalum, and B. clonalum. However, the predictive ability of OAS3 was similar to that of IFGN (AUC>0.5 for 17 ICD subgroups), but lower than that of CD274, TIDE and Merk18. In addition, the results demonstrated that high expression levels of *OAS3* were associated with better PD1 OS in melanoma (Gide2019_PD1, Liu2019_PD1) and better PFS in kidney renal clear cell carcinoma and melanoma (Miao2018_ICB, Liu2019_PD1) (Figures 8B, C).

### Pathway Analysis

We performed Spearman correlation analysis on *OAS3* and pathway scores. A close association was observed between *OAS3* and pathways including tumour inflammation signature, cellular response to hypoxia, tumour proliferation signature, angiogenesis, and apoptosis. The higher the expression of *OAS3*, the stronger the activity of the related pathway (Supplementary Figure S10).

## Discussion

The molecular mechanisms underlying the role of *OAS3* in the immune microenvironment and pathogenesis of different tumours remain unknown. In this study, we performed an integrative analysis of molecular characteristics, oncogenic roles and relevant immune and pharmacogenomic features of *OAS3* in pan-cancer. The findings suggest that *OAS3* is closely associated with the development of various systemic diseases and cancers. In addition, a functional association was observed between *OAS3* and TME, especially immunosuppressive cells.

We compared the expression of *OAS3* in normal and tumour tissues of 33 cancers and found that *OAS3* was dysregulated and highly expressed in almost all TCGA tumour types and was associated with the staging or metastasis of DLBC, HNSC, KIRC, LIHC, LUSC, MESO, OV, PAAD, LUAD, SKCM, and UCS. These findings suggest that OAS3 is an important regulator of carcinogenesis, progression, invasion and metastasis in various cancers. Regarding the prognostic value of *OAS3* in pan-cancer, we observed that *OAS3* was closely associated with survival indicators such as OS, DSS, DFS, and PFS, and high *OAS3* expression was associated with poorer survival rates in CESC, GBM, KICH, KIRP, LAML, LGG, LIHC, LUAD, LUSC, PAAD, TGCT, and ACC. In previous studies, *OAS3* has been reported as a risk factor for different cancers, which is consistent with the results of this study (Piera-Velazquez et al., 2021; Calvet et al., 2022; Shi et al., 2022).

TME is closely associated with tumour growth, progression and prognosis and immune cells play an important role in tumour growth and progression (Lei et al., 2021). Previous studies have found that cytokines in TME regulate immune function and eventually suppress the immune response, leading to tumour progression (Hinshaw and Shevde, 2019). Therefore, analysing the components of TME can help to develop drugs for targeted tumour immunotherapy. In this study, a significant positive correlation was observed between *OAS3* and the immunosuppressive cells iTregs and nTregs. Immune checkpoint inhibitors are effective anti-cancer immunotherapeutic approaches (Li et al., 2019). In this study, *OAS3* expression was positively correlated with 47 immune checkpoint genes in most cancer types. Therefore, *OAS3* can be used as a novel drug target for anti-cancer immunotherapy.

Furthermore, we analysed the correlation between the expression of *OAS3* and MMR-related genes, RNA methylation genes and four DNA methyltransferases. MMR can repair errors that occur during DNA replication (Vilar and Gruber, 2010) and is known to repair microsatellite replication errors. However, defects in MMR (dMMR) can lead to MSI (Jiricny, 2006). RNA methylation plays a crucial role in the tumorigenesis and progression of tumours (Chen et al., 2020). Altered DNA methylation is also associated with tumorigenesis (Kulis and Esteller, 2010). Based on the findings of this study, *OAS3* expression is positively correlated with MMR-related genes, RNA methylation and four DNA methyltransferases in most cancers. Altogether, the findings demonstrate that *OAS3* mediates tumorigenesis by regulating DNA damage and DNA and RNA methylation.

Based on the abovementioned results, *OAS3* may serve as a very important biomarker for tumour immunotherapy. Monoclonal antibodies-targeting CTLA-4, PD-1 or PDL-1 have shown clinical potential for effectively controlling and treating human cancers (Liu et al., 2021b). This study showed that patients with melanoma and kidney renal clear cell carcinoma with high *OAS3* expression had a higher clinical benefit from ICB treatment (PD-1 or PD-L1). On analysing the relationship between *OAS3* and the IC50 of drugs using the GDSC2 database, we found that high *OAS3* expression might lead to resistance to BI-2536, GSK269962A, vorinostat, sorafenib, and BMS-754807. However, high *OAS3* expression was negatively correlated with trametinib, sapitinib, SCH772984, selumetinib, and dasatinib. This finding provides a basis for selecting anti-tumour agents for patients in the future.

## Conclusion

This study showed that *OAS3* is highly expressed in various tumours, and high *OAS3* expression is associated with poor survival outcomes. In addition, we demonstrated the association between *OAS3* and the expression of immune-infiltrating cells, immune checkpoint genes, TMB, and MSI. *OAS3* may influence tumour progression through immunosuppression. The evidence for the significant immunological utility of *OAS3* as a prognostic and immunotherapeutic biomarker for pan-cancer provides compelling new insights into the potential development of future immunotherapeutic and diagnostic trials. Therefore, the findings of this study will contribute to the development of new therapeutic approaches for patients with cancer, improving their treatment and prognosis.

## Data Availability

The datasets presented in this study can be found in online repositories. The names of the repository/repositories and accession number(s) can be found in the article/Supplementary Material.

## Supplementary Material

Supplementary Figure 1 Immunofluorescence staining of the subcellular distribution of OAS3 from the HPA database. We observed that OAS3 colocalised with ER and microtubule markers in A431, A549, and U-2 OS cells, suggesting the subcellular localisation of OAS3 in ER and microtubules.

Supplementary Figure 2 Boxplots showing differential OAS3 expression levels (log2 TPM + 1) between pathological stages and DLBC, HNSC, KIRC, LIHC, LUSC, MESO, OV, PAAD, LUAD, SKCM, and UCS were positively correlated with the expression of OAS3. This finding suggests that OAS3 plays an important role in tumorigenesis. Only TCGA cancers with statistically significant differences between the pathological stages are presented.

Supplementary Figure 3 Kaplan-Meier analysis for OS according to OAS3 expression. Only statistically significant cancers (*p*-value < 0.05) are listed.

Supplementary Figure 4 Kaplan-Meier analysis for DFS according to OAS3 expression. Only statistically significant cancers (*p*-value < 0.05) are listed.

Supplementary Figure 5 Kaplan-Meier analysis for DSS according to OAS3 expression. Only statistically significant cancers (*p*-value < 0.05) are listed.

Supplementary Figure 6 The correlation between OAS3 and immune scores **(A)**, ESTIMATEScore **(B)**, and stromal scores **(C)** in pancancer.

Supplementary Figure 7 Four methods were used to calculate the correlation between OAS3 and immune-related cells. Xcell **(A)**, TIMER2 **(B)**, CIBERSORT **(C)**, and ImmuCellAI **(D)**. The lower upper triangle of each square represented the magnitude of the p value of the correlation test, and the upper triangle represented the correlation coefficient (∗*p* < 0.05, ∗∗*p* < 0.01, ∗∗∗*p* < 0.001).

Supplementary Figure 8
**(A)** OAS3 had a significant positive correlation with RNA methylation-related genes (m1A, m5C, and m6A) in most tumours. The lower upper triangle of each square represented the magnitude of the *p* value of the correlation test, and the upper triangle represented the correlation coefficient (∗p < 0.05, ∗∗*p* < 0.01, ∗∗∗*p* < 0.001). **(B)** A positive correlation was found between OAS3 and four immune pathway-related genes (receptor, MHC, immunoinhibitors, and immunostimulators) in many tumour types. The lower upper triangle of each square represented the magnitude of the p value of the correlation test, and the upper triangle represented the correlation coefficient (∗*p* < 0.05, ∗∗*p* < 0.01, ∗∗∗*p* < 0.001).

Supplementary Figure 9 The correlation between the sensitivity of drugs in the Genomic of Drug Sensitivity in Cancer (GDSC) database **(A)**, CTRP database **(B)**, and mRNA expression of OAS3. The Pearson’s correlation indicates the correlation between gene expression and drugs sensitivity. Blue bubbles represented negative correlations, and red bubbles represented positive correlations; the deeper the color, the higher the correlation. The bubble size was positively correlated with the FDR significance.

Supplementary Figure 10 Pathway analysis. The abscissa represents the distribution of the gene expression, and the ordinate represents the distribution of the pathway score. The density curve on the right represents the trend in distribution of pathway immune score, the upper density curve represents the trend in distribution of the gene expression. The value on the top represents the correlation *p* value, correlation coefficient and correlation calculation method.

## References

[B1] Alexandrov L. B. Nik-Zainal S. Wedge D. C. Aparicio S. A. Behjati S. Biankin A. V. (2013). Signatures of Mutational Processes in Human Cancer. Nature 500 (7463), 415–421. 10.1038/nature12477 23945592 PMC3776390

[B2] Alhopuro P. Sammalkorpi H. Niittymäki I. Biström M. Raitila A. Saharinen J. (2012). Candidate Driver Genes in Microsatellite-Unstable Colorectal Cancer. Int. J. Cancer 130 (7), 1558–1566. 10.1002/ijc.26167 21544814

[B3] Calvet J. Berenguer-Llergo A. Gay M. Massanella M. Domingo P. Llop M. (2022). Biomarker Candidates for Progression and Clinical Management of COVID-19 Associated Pneumonia at Time of Admission. Sci. Rep. 12 (1), 640. 10.1038/s41598-021-04683-w 35022497 PMC8755735

[B4] Chen J. Yu K. Zhong G. Shen W. (2020). Identification of a m6A RNA Methylation Regulators-Based Signature for Predicting the Prognosis of clear Cell Renal Carcinoma. Cancer Cel Int 20, 157. 10.1186/s12935-020-01238-3 PMC7206820 32419773

[B5] Chen Y.-J. Liao W.-X. Huang S.-Z. Yu Y.-F. Wen J.-Y. Chen J. (2021). Prognostic and Immunological Role of CD36: A Pan-Cancer Analysis. J. Cancer 12 (16), 4762–4773. 10.7150/jca.50502 34234847 PMC8247371

[B6] Chen Y. Meng Z. Zhang L. Liu F. (2021). CD2 Is a Novel Immune-Related Prognostic Biomarker of Invasive Breast Carcinoma that Modulates the Tumor Microenvironment. Front. Immunol. 12, 664845. 10.3389/fimmu.2021.664845 33968066 PMC8102873

[B7] Coppedè F. Lopomo A. Spisni R. Migliore L. (2014). Genetic and Epigenetic Biomarkers for Diagnosis, Prognosis and Treatment of Colorectal Cancer. Wjg 20 (4), 943–956. 10.3748/wjg.v20.i4.943 24574767 PMC3921546

[B8] Ellegren H. (2004). Microsatellites: Simple Sequences with Complex Evolution. Nat. Rev. Genet. 5 (6), 435–445. 10.1038/nrg1348 15153996

[B9] Grady W. M. Yu M. Markowitz S. D. (2021). Epigenetic Alterations in the Gastrointestinal Tract: Current and Emerging Use for Biomarkers of Cancer. Gastroenterology 160 (3), 690–709. 10.1053/j.gastro.2020.09.058 33279516 PMC7878343

[B10] Grauel A. L. Nguyen B. Ruddy D. Laszewski T. Schwartz S. Chang J. (2020). Tgfβ-Blockade Uncovers Stromal Plasticity in Tumors by Revealing the Existence of a Subset of Interferon-Licensed Fibroblasts. Nat. Commun. 11 (1), 6315. 10.1038/s41467-020-19920-5 33298926 PMC7725805

[B11] Hinshaw D. C. Shevde L. A. (2019). The Tumor Microenvironment Innately Modulates Cancer Progression. Cancer Res. 79 (18), 4557–4566. 10.1158/0008-5472.CAN-18-3962 31350295 PMC6744958

[B12] Huang C. Jiang X. Huang Y. Zhao L. Li P. Liu F. (2021). Identifying Dendritic Cell-Related Genes through a Co-expression Network to Construct a 12-Gene Risk-Scoring Model for Predicting Hepatocellular Carcinoma Prognosis. Front. Mol. Biosci. 8, 636991. 10.3389/fmolb.2021.636991 34109210 PMC8181399

[B13] Jiang Y. Li Y. Zhu B. (2015). T-cell Exhaustion in the Tumor Microenvironment. Cell Death Dis 6, e1792. 10.1038/cddis.2015.162 26086965 PMC4669840

[B14] Jiricny J. (2006). The Multifaceted Mismatch-Repair System. Nat. Rev. Mol. Cel Biol 7 (5), 335–346. 10.1038/nrm1907 16612326

[B15] Ju Q. Li X. Zhang H. Yan S. Li Y. Zhao Y. (2020). NFE2L2 Is a Potential Prognostic Biomarker and Is Correlated with Immune Infiltration in Brain Lower Grade Glioma: A Pan-Cancer Analysis. Oxidative Med. Cell. longevity 2020, 1–26. 10.1155/2020/3580719 PMC7569466 33101586

[B16] Khanna K. Jamwal V. L. Kohli S. K. Gandhi S. G. Ohri P. Bhardwaj R. (2019). Plant Growth Promoting Rhizobacteria Induced Cd Tolerance in Lycopersicon esculentum through Altered Antioxidative Defense Expression. Chemosphere 217, 463–474. 10.1016/j.chemosphere.2018.11.005 30445394

[B17] Kulis M. Esteller M. (2010). DNA Methylation and Cancer. Adv. Genet. 70, 27–56. 10.1016/B978-0-12-380866-0.60002-2 20920744

[B18] Lei K. Li J. Tu Z. Liu F. Ye M. Wu M. (2021). Prognostic and Predictive Value of Immune-Related Gene Pair Signature in Primary Lower-Grade Glioma Patients. Front. Oncol. 11, 665870. 10.3389/fonc.2021.665870 34123829 PMC8190397

[B19] Li B. Chan H. L. Chen P. (2019). Immune Checkpoint Inhibitors: Basics and Challenges. Cmc 26 (17), 3009–3025. 10.2174/0929867324666170804143706 28782469

[B20] Liao J.-Y. Zhang S. (2021). Safety and Efficacy of Personalized Cancer Vaccines in Combination with Immune Checkpoint Inhibitors in Cancer Treatment. Front. Oncol. 11, 663264. 10.3389/fonc.2021.663264 34123821 PMC8193725

[B21] Lin Q. H. Zhang K. D. Duan H. X. Liu M. X. Wei W. L. Cao Y. (2015). ERGIC 3, Which Is Regulated by miR‐203a, Is a Potential Biomarker for Non‐small Cell Lung Cancer. Cancer Sci. 106 (10), 1463–1473. 10.1111/cas.12741 26177443 PMC4638005

[B22] Liu C. Zhang G. Xiang K. Kim Y. Lavoie R. R. Lucien F. (2021). Targeting the Immune Checkpoint B7-H3 for Next-Generation Cancer Immunotherapy. Cancer Immunol Immunother 11, 654684. 10.1007/s00262-021-03097-x PMC10991665 34739560

[B23] Liu S. Liang J. Liu Z. Zhang C. Wang Y. Watson A. H. (2021). The Role of CD276 in Cancers. Front. Oncol. 11, 654684. 10.3389/fonc.2021.654684 33842369 PMC8032984

[B24] Liu Y. Cao X. (2016). Immunosuppressive Cells in Tumor Immune Escape and Metastasis. J. Mol. Med. 94 (5), 509–522. 10.1007/s00109-015-1376-x 26689709

[B25] Luo Q. Vögeli T.-A. (2020). A Methylation-Based Reclassification of Bladder Cancer Based on Immune Cell Genes. Cancers 12 (10), 3054. 10.3390/cancers12103054 PMC7593922 33092083

[B26] Mcelhinney J. M. W. R. Hasan A. Sajini A. A. (2020). The Epitranscriptome Landscape of Small Noncoding RNAs in Stem Cells. Stem cells (Dayton, Ohio) 38 (10), 1216–1228. 10.1002/stem.3233 PMC7586957 32598085

[B27] Min A. Kim K. Jeong K. Choi S. Kim S. Suh K. J. (2020). Homologous Repair Deficiency Score for Identifying Breast Cancers with Defective DNA Damage Response. Sci. Rep. 10 (1), 12506. 10.1038/s41598-020-68176-y 32719318 PMC7385153

[B28] Monteran L. Erez N. (2019). The Dark Side of Fibroblasts: Cancer-Associated Fibroblasts as Mediators of Immunosuppression in the Tumor Microenvironment. Front. Immunol. 10, 1835. 10.3389/fimmu.2019.01835 31428105 PMC6688105

[B29] Petrelli F. Ghidini M. Cabiddu M. Pezzica E. Corti D. Turati L. (2019). Microsatellite Instability and Survival in Stage II Colorectal Cancer: A Systematic Review and Meta-Analysis. Anticancer Res. 39 (12), 6431–6441. 10.21873/anticanres.13857 31810907

[B30] Piera-Velazquez S. Mendoza F. A. Addya S. Pomante D. Jimenez S. A. (2021). Increased Expression of Interferon Regulated and Antiviral Response Genes in CD31+/CD102+ Lung Microvascular Endothelial Cells from Systemic Sclerosis Patients with End-Stage Interstitial Lung Disease. Clin. Exp. Rheumatol. 39 (6), 1298–1306. 33253099 10.55563/clinexprheumatol/ret1kg

[B31] Popp H. D. Bohlander S. K. (2010). Genetic Instability in Inherited and Sporadic Leukemias. Genes Chromosom. Cancer 49 (12), 1071–1081. 10.1002/gcc.20823 20842730

[B32] Schulze A. Oshi M. Endo I. Takabe K. (2020). MYC Targets Scores Are Associated with Cancer Aggressiveness and Poor Survival in ER-Positive Primary and Metastatic Breast Cancer. Ijms 21 (21), 8127. 10.3390/ijms21218127 PMC7663719 33143224

[B33] Shi Y. Xu Y. Xu Z. Wang H. Zhang J. Wu Y. (2022). TKI Resistant-Based Prognostic Immune Related Gene Signature in LUAD, in Which FSCN1 Contributes to Tumor Progression. Cancer Lett. 532, 215583. 10.1016/j.canlet.2022.215583 35149175

[B34] Thommen D. S. Schumacher T. N. (2018). T Cell Dysfunction in Cancer. Cancer cell 33 (4), 547–562. 10.1016/j.ccell.2018.03.012 29634943 PMC7116508

[B35] Tian S. Lai J. Yu T. Li Q. Chen Q. (2021). Regulation of Gene Expression Associated with the N6-Methyladenosine (m6A) Enzyme System and its Significance in Cancer. Front. Oncol. 10, 623634. 10.3389/fonc.2020.623634 33552994 PMC7859513

[B36] Tomczak K. Czerwińska P. Wiznerowicz M. (2015). Review the Cancer Genome Atlas (TCGA): an Immeasurable Source of Knowledge. wo 1A (1A), 68–77. 10.5114/wo.2014.47136 PMC4322527 25691825

[B37] Toyota M. Suzuki H. (2010). Epigenetic Drivers of Genetic Alterations. Adv. Genet. 70, 309–323. 10.1016/B978-0-12-380866-0.60011-3 20920753

[B38] Vilar E. Gruber S. B. (2010). Microsatellite Instability in Colorectal Cancer-The Stable Evidence. Nat. Rev. Clin. Oncol. 7 (3), 153–162. 10.1038/nrclinonc.2009.237 20142816 PMC3427139

[B39] Wu Z. Zhu K. Liu Q. Liu Y. Chen L. Cui J. (2020). Profiles of Immune Infiltration in Bladder Cancer and its Clinical Significance: an Integrative Genomic Analysis. Int. J. Med. Sci. 17 (6), 762–772. 10.7150/ijms.42151 32218698 PMC7085262

[B40] Xu C. Zang Y. Zhao Y. Cui W. Zhang H. Zhu Y. (2021). Comprehensive Pan-Cancer Analysis Confirmed that ATG5 Promoted the Maintenance of Tumor Metabolism and the Occurrence of Tumor Immune Escape. Front. Oncol. 11, 652211. 10.3389/fonc.2021.652211 33842365 PMC8027486

[B41] Yang M. Huang W. Sun Y. Liang H. Chen M. Wu X. (2019). Prognosis and Modulation Mechanisms of COMMD6 in Human Tumours Based on Expression Profiling and Comprehensive Bioinformatics Analysis. Br. J. Cancer 121 (8), 699–709. 10.1038/s41416-019-0571-x 31523056 PMC6889128

[B42] Yang Y. Deng X. Chen X. Chen S. Song L. Meng M. (2020). Landscape of Active Enhancers Developed De Novo in Cirrhosis and Conserved in Hepatocellular Carcinoma. Am. J. Cancer Res. 10 (10), 3157–3178. 33163263 PMC7642653

[B43] Yuan F. Ming H. Wang Y. Yang Y. Yi L. Li T. (2020). Molecular and Clinical Characterization of Galectin‐9 in Glioma through 1,027 Samples. J. Cel Physiol 235 (5), 4326–4334. 10.1002/jcp.29309 PMC7028024 31609000

[B44] Zhang Y. Yu C. (2020). Prognostic Characterization of OAS1/OAS2/OAS3/OASL in Breast Cancer. BMC cancer 20 (1), 575. 10.1186/s12885-020-07034-6 32560641 PMC7304174

[B45] Zhou R. Zeng D. Zhang J. Sun H. Wu J. Li N. (2019). A Robust Panel Based on Tumour Microenvironment Genes for Prognostic Prediction and Tailoring Therapies in Stage I-III colon Cancer. EBioMedicine 42, 420–430. 10.1016/j.ebiom.2019.03.043 30917936 PMC6491960

